# From beliefs to behavior: Clarifying the roles of attitudes and context

**DOI:** 10.1002/jcpy.70012

**Published:** 2025-12-01

**Authors:** Hogeun Lee, Dolores Albarracín

**Affiliations:** University of Pennsylvania, Philadelphia, Pennsylvania, USA

**Keywords:** BDT, beliefs and lay theories, persuasion

## Abstract

Two commentaries by Tormala and Rucker and by Critcher and Galak offer complementary perspectives on our target article, *Changing Beliefs* versus *Changing Behavior*. Tormala and Rucker emphasize attitudes as proximal determinants of behavior, underscoring the importance of attitude strength and measurement compatibility. Critcher and Galak propose a contextual continuum of decision making that situates belief influence within unprompted, opportunity-based, and prompted behavioral contexts, illustrating how contextual factors shape the recruitment of beliefs for specific actions. In this response, we clarify the relations among beliefs, attitudes, and context; address the misconception that our model overemphasizes inferential reasoning; and extend the framework to integrate evaluative and contextual determinants of behavior. This synthesis offers a comprehensive account in which attitudes function as inferential products of belief processing, while contexts determine when and how these processes are activated.

In *Changing Beliefs* versus *Changing Behavior*, we proposed a framework to explain why changes in belief do not always lead to changes in behavior, identify the conditions under which beliefs translate into behaviors, and outline effective interventions for correcting beliefs versus modifying behaviors. Grounded in the belief-to-behavior inference model (Granados Samayoa & Albarracín, 2025), the framework posits that beliefs are more likely to influence behavior when three conditions are met: (a) a behavioral goal is activated, (b) the inferential path from belief to behavior is short, and (c) the belief–behavior connection is preserved in memory or activated in the moment. These conditions provide a cognitive architecture for translating beliefs into action, helping to explain why belief–behavior correlations are often modest and context-dependent (Albarracín et al., 2024; Granados Samayoa & Albarracín, 2025).

The two commentaries published alongside our target article offer valuable extensions to this framework. Tormala and Rucker (2025) draw attention to attitudes as proximal predictors of behavior and highlight established factors that influence the strength of the attitude–behavior link, such as attitude strength and measurement compatibility. Critcher and Galak (2025) shift the focus to environmental determinants, arguing that belief–behavior correspondence is most likely to emerge when decision contexts prompt belief recruitment, rather than when individuals must infer behavior from beliefs in unprompted situations. Both analyses are insightful and together broaden the scope of our framework.

We concur with both commentaries that the belief–behavior relation cannot be reduced to a single pathway. Rather, it reflects a dynamic interplay among cognitive representations, motivational orientations, and contextual affordances. The purpose of this article is to summarize the commentaries and clarify how each aligns with the belief-to-behavior inference model. We do so by identifying key points of convergence and divergence, addressing misinterpretations, and incorporating attitude strength and decision context within the practical domain of misinformation correction.

## RESPONSE TO TORMALA AND RUCKER

### Attitudes in the belief-to-behavior inference framework

We agree with Tormala and Rucker (2025) that attitudes are proximal determinants of behavior and that the extensive literature on the attitude–behavior relation (Ajzen & Fishbein, 1980; Glasman & Albarracín, 2006) provides indispensable insights. We also concur that, because the same belief can yield different evaluative judgments, measuring attitudes is essential for identifying why a given belief may fail to produce corresponding behavior. This view aligns with our definition of beliefs and attitudes and the role of belief-based inferences: beliefs represent cognitive probability judgments linking an entity–to an attribute or outcome, whereas attitudes constitute evaluative judgments along a good–bad dimension (Granados Samayoa & Albarracín, 2025).

Although our target article did not explicitly discuss the role of attitudes, the belief-to-behavior inference model incorporates behavioral attitudes as integral components of the inferential chain linking beliefs to behavior (Granados Samayoa & Albarracín, 2025). As we argued in our target article (Lee & Albarracín, 2025), belief-to-behavior inferences are forms of practical reasoning that connect a descriptive or outcome belief to a behavioral decision through an inferential process that goes through behavioral attitudes and behavioral intention. This reasoning cannot proceed without an evaluative warrant that justifies behavior. In other words, every syllogistic reasoning that moves from belief to behavior entails an evaluative step, which is equivalent to attitudes and is explicitly discussed as such in Granados Samayoa and Albarracín (2025).

The model’s discussion of proceduralization also intersects directly with the literature on automatic activation attitudes. Once a belief-to-behavior inference is repeatedly enacted, its evaluative component can become stored independently from the initial belief that produced it. As we noted, attitudes, intentions, and behaviors can later be activated even when the original belief has been forgotten (Granados Samayoa & Albarracín, 2025). This account parallels classic findings on attitude strength and accessibility (Bargh et al., 2001; Glasman & Albarracín, 2006; Krosnick & Petty, 1995; Tormala & Rucker, 2018): the evaluative residue of prior inference can continue to shape behavior long after the initial belief is no longer salient. In this way, the belief-to-behavior inference converges by acknowledging that evaluative representations are part of the belief-to-behavior inference and can persist as autonomous guides for future behavior.

### Attitudes and behavioral goals

The core of our theoretical framework lies in the *role of behavioral goals*. As emphasized in our target article, beliefs influence behavior only when motivational factors direct reasoning toward a specific behavioral decision (Granados Samayoa & Albarracín, 2025; Lee & Albarracín, 2025). In the absence of such a goal, even substantial belief change is unlikely to produce action. The same principle applies to attitudes: without a salient behavioral goal, evaluative judgments remain inert. As we discussed when introducing strategies that shift cognitive feelings, even though beliefs and attitudes may change through associative or metacognitive processes (Petty et al., 2013; Staats & Staats, 1958; Zajonc, 1968), these changes lack behavioral relevance until they are retrieved in contexts where the behavioral goal is salient.

More importantly, as attitudes can be *goal-dependent*, their valence and meaning vary according to the behavioral context under consideration. Drawing from Tormala and Rucker’s (2025) condominium example, if an individual’s goal is to purchase and live permanently in the property, the belief that the condo is “quiet” may elicit a negative evaluation among buyers seeking an active nightlife. Conversely, if the goal is to rent the same condo temporarily for relaxation, that identical belief produces a positive evaluation. This illustration reveals that evaluative meaning emerges from the conjunction of belief content and behavioral goal. In the terminology of the belief-to-behavior inference model, the behavioral goal determines the evaluative criteria by which a belief is appraised, shaping the specific behavioral attitudes and, consequently, the behavioral decisions. Beliefs, behavioral attitudes, and behaviors are thus dynamically interdependent, yet their correspondence ultimately depends on which behavioral goal is salient.

This argument aligns with the importance of measurement. The correspondence between beliefs, attitudes, intentions, and behaviors depends on their alignment in *time, action, context, and target* (Fishbein & Ajzen, 1975, 2010). Although our target article did not focus on measurement per se, it indirectly captures this principle through the *length of the inferential chain*: the greater the mismatch in specificity, the longer the chain connecting belief to behavior, and the greater the likelihood that the process halts before reaching action (Granados Samayoa & Albarracín, 2025; Lee & Albarracín, 2025). When beliefs directly reference the specific behavior of interest, their inferential distance is short and their impact on behavior is strong. When beliefs are abstract or temporally misaligned, the inferential distance lengthens and their influence wanes. Thus, measurement compatibility and inferential distance reflect measurement and process instances of the same principle—alignment determines whether belief reasoning successfully bridges cognition and behavior.

### Roles of social norms and perceived behavioral control

Last but not least, we agree with Tormala and Rucker (2025) that social norms and perceived behavioral control are important determinants of behavior. We emphasized this in the target article (Lee & Albarracín, 2025, p. 8) that “[O]nce attitudes and intentions govern behavior decoupled from belief in memory, belief correction alone will be moot. Effective interventions may still be possible at that stage, but will require interventions that address attitudes, social norms, habits, and other immediate behavioral precursors.”

We contend that our unique contribution to the misinformation literature lies in extending the focus beyond belief and attitude change to include interventions that explicitly address beliefs and those targeting the social and structural determinants of behavior. Whereas prior misinformation research has primarily emphasized correcting false beliefs and altering attitudes, our review (Lee & Albarracín, 2025) systematically catalogued strategies that target social norms and perceived control. In the section on social-structural interventions, we highlighted how mechanisms such as normative messaging, social support, and environmental access can facilitate behavior even when belief change alone is insufficient. In this way, our contribution bridges the gap between cognitive and contextual approaches, situating misinformation interventions within a multilevel framework that links individual reasoning to the social and institutional conditions that enable belief- and attitude-consistent action.

## RESPONSE TO CRITCHER AND GALAK

### Clarification on the length of the inferential chain

Critcher and Galak (2025) rightly caution against equating small correlations between a belief and a behavior with weak causal influence. They argue that multiple beliefs often compete for influence and that a single belief can inform multiple behaviors. We agree with this and maintain that the key inquiry is not whether beliefs affect behavior, but rather *which* beliefs do so, *under what conditions*, and *in what contexts*. They also concur with our argument that beliefs connected to behavior through shorter inferential chains are more likely to translate into action, yet they propose an alternative mechanism for this same result: synthesized, summary beliefs may exert greater behavioral impact than individual distal beliefs, which must compete with one another to influence behavior as they pass through the decision-making funnel (Critcher & Galak, 2025).

While we acknowledge that this is a valid insight, we wish to clarify that our concept of inferential distance differs from their interpretation. The length of the inferential chain does not correspond to the stage of a decision-making funnel but reflects the number of logical steps required to derive a relevant behavioral conclusion from a given belief. To illustrate this distinction, we turn to the same example of hotel service that they employed. In our formulation, an individual belief such as “Our room was never serviced during our weeklong stay” and a summary belief such as “This hotel does not offer consistent service” have the same inferential length. Both are descriptive beliefs, regardless of their level of abstraction. To shorten the inferential chain, an outcome belief is required. For instance, a customer may have an individual descriptive belief, “The towels and linens were never replaced,” which falls under the service category. A corresponding outcome belief might be, “Staying in this hotel will affect my health,” which can directly yield an evaluation, “Something that causes skin trouble is bad,” and consequently the attitudinal and behavioral conclusion, “If the hotel is bad, I will not stay here again.” This difference in framework yields distinct predictions. We predict that an individual outcome belief will exert a stronger influence on behavior than a summary descriptive belief, despite the latter being more general and positioned closer to behavior within the decision-making funnel.

### Practical reasoning, opportunities and prompts, and behavioral goals

While Critcher and Galak (2025) acknowledge that belief-to-behavior inferences can indeed be formed— and that, once formed, they exert a powerful influence by overriding competing beliefs—they argue that such cases are atypical. They note that people typically do not engage in this kind of logical reasoning, and this account differs from prior theories explaining how beliefs influence behavior (Ajzen, 2011; Anderson, 1974; Fishbein & Ajzen, 1975, 2010; Greenwald et al., 2009). We agree with this critique and wish to clarify that the belief-to-behavior inference model does not claim that *logical* reasoning represents the typical mode of human processing. Rather, the model seeks to explain the circumstances under which a single belief can effectively guide behavior through *practical* reasoning (e.g., reasoning about what is good to do). In the absence of practical inferential reasoning, beliefs remain cognitively isolated—stored as independent representations that are disconnected from downstream constructs such as attitudes, intentions, and behaviors (Granados Samayoa & Albarracín, 2025; Lee & Albarracín, 2025). In this view, beliefs can exist as passive cognitive elements until they are actively incorporated into a reasoning process that links them to action.

This is where Critcher and Galak’s (2025) framework and ours converge. We agree that belief-to-behavior inferences rarely occur spontaneously from the formation of a belief alone. They require the activation of a behavioral goal that motivates individuals to consider how a given belief relates to a potential action. In the absence of such a goal, beliefs remain cognitively isolated and detached from behaviorally relevant processes (Granados Samayoa & Albarracín, 2025; Lee & Albarracín, 2025). In this regard, what Critcher and Galak (2025) describe as behavioral opportunities and prompted decisions directly correspond to what we conceptualize as contexts that activate behavioral goals—moments when belief reasoning becomes behaviorally operative.

Therefore, the two frameworks capture different segments of the same process. Critcher and Galak (2025) emphasize the situational conditions that elicit belief recruitment, whereas our framework focuses on the cognitive mechanisms that form links between beliefs and behavior. Prompts and opportunities represent the external triggers of the same motivational process that our model identifies as goal activation. In this sense, the two accounts are complementary rather than conflicting. Their emphasis on behavioral prompts and opportunities provides concrete predictions about which contexts are most likely to activate behavioral goals, while our model explains which beliefs are more likely to be recruited once those goals are triggered. Specifically, beliefs with shorter inferential chains or those that have become proceduralized are more likely to be retrieved and to exert influence on behavioral decisions (Granados Samayoa & Albarracín, 2025; Lee & Albarracín, 2025).

### Implications for methodologies and practical application

Critcher and Galak (2025) argue that researchers often treat the presence or absence of behavioral outcomes as a definitive test of theoretical relevance, without fully considering the decision contexts in which those outcomes are observed. When laboratory studies include direct prompts—such as requiring participants to report behavioral intentions—they effectively engineer conditions suited to prompted decisions. Within our framework, such environments are likely to elicit belief-to-behavior inferences because the belief modified by the experimental stimulus becomes salient and thus behaviorally relevant in that moment. Consequently, these designs may overestimate the apparent impact of belief change on behavior.

Critcher and Galak (2025) make an excellent point that the behavioral relevance of a belief cannot be evaluated in isolation from the circumstances that elicit its use. We also agree with their reflections on how psychologists should approach the study of belief and behavior. However, we would add that even though experimental conditions of limited external validity may inflate belief–behavior correspondence, the observed relation is not merely a methodological artifact. The relation is genuine, but is diluted in real-world contexts where behavioral prompts and goal activation are less likely.

With this in mind, the question shifts to how researchers and interventionists can make a given belief more salient at the time and place most proximal to actual decision-making. In other words, if researchers can “engineer” behavioral outcomes in laboratory settings through methodological design, similar principles can and should be applied by practitioners developing real-world interventions. As illustrated in our target article (Lee & Albarracín, 2025, p. 13), one practical example involves delivering corrective messages at moments directly tied to the targeted behavior. For instance, information that counters misinformation about mask-wearing may be presented at the entrance of a public building, rather than airing the same message on television in the middle of the night. This approach ensures that beliefs are not only corrected but also rendered behaviorally consequential at the point of decision.

## CONCLUSION

Both commentaries significantly enrich the discussion initiated in *Changing Beliefs* versus *Changing Behavior* (Lee & Albarracín, 2025). Tormala and Rucker (2025) advanced our framework by emphasizing attitudes as proximal determinants of behavior and by underscoring the importance of attitude strength and measurement compatibility. Their commentary allowed us to clarify how evaluative components are embedded within belief-to-behavior inferences and to further articulate the role of behavioral goals not only in linking beliefs to behaviors but also in shaping the belief–attitude relation. This dialogue also provided an opportunity to reaffirm that our broader framework accounts for other factors, including social norms and structural factors that jointly determine when belief and attitude change translate into action.

Critcher and Galak (2025) extended our framework by situating belief reasoning within the decision contexts, providing a complement to our emphasis on cognitive and motivational mechanisms. Through their commentary, we were able to clarify that the belief-to-behavior inference model does not portray logical reasoning as the default mode of human thought but rather practical reasoning as a necessary condition for a belief to guide action when a behavioral goal is present. Their framework also prompted us to specify how contextual cues such as decision prompts function as external activators of behavioral goals, thereby aligning with the internal mechanisms we described. Altogether, their contribution helped us articulate how contextual, motivational, and social factors converge to determine when and why beliefs become behaviorally consequential.

Revisiting these insights about misinformation and behavioral change underscores the broader relevance of this theoretical synthesis. Belief correction alone rarely alters behavior unless accompanied by the activation of behavioral goals, supportive social norms, and contextual opportunities to act. Effective interventions must therefore operate at multiple levels, targeting the inferential pathways that connect beliefs to behaviors, reinforcing evaluative clarity through attitude formation, and structuring environments that cue behavioral decisions at the right time and place. In this sense, both commentaries reinforce our central claim: meaningful behavior change depends not merely on what people believe, but on when, where, and how those beliefs are mobilized.

We are grateful to the commentators for extending our framework and for helping to clarify the cognitive, motivational, and contextual forces that determine when belief change becomes behavioral change. Their contributions advance not only theoretical precision but also the practical science of combating misinformation and fostering accurate, belief-consistent behavior in the real world.

## Data Availability

Data sharing not applicable to this article as no datasets were generated or analysed during the current study.
